# Information-theoretic multi-scale geometric pre-training for enhanced molecular property prediction

**DOI:** 10.1371/journal.pone.0332640

**Published:** 2025-10-06

**Authors:** Xiaoyu Hu, Xiuyuan Zhao, Jiyuan Wang, Yongbin Yang

**Affiliations:** 1 Department of Chemical Engineering and Materials Science, Stevens Institute of Technology, Hoboken, New Jersey, United States of America; 2 Stevens Institute of Technology, Hoboken, New Jersey, United States of America; 3 Duke University, Durham, North Carolina, United States of America; 4 University of Southern California, Los Angeles, California, United States of America; NED University of Engineering and Technology, PAKISTAN

## Abstract

Maximizing information transfer across different structural scales is critical for effective molecular representation learning. Current molecular graph neural networks fail to fully capture the multi-scale nature of molecular geometry, leading to suboptimal information propagation between local and global structural features. We propose Multi-Scale Geometric Pre-training (MSG-Pre), an information-theoretic framework that hierarchically integrates molecular information across atomic, functional group, and conformer levels through entropy-guided mechanisms. Our approach employs a scale-adaptive attention mechanism that dynamically weights geometric features based on their information content, coupled with a hierarchical contrastive learning scheme that maximizes mutual information between complementary structural views. This is further reinforced by a geometric regularization strategy that minimizes information loss of essential conformational properties. Rigorous empirical validation on 14 molecular benchmark datasets demonstrates state-of-the-art performance with improvements up to 5.2% over previous methods. Notably, MSG-Pre significantly enhances information extraction for nanomedicine applications including nanoparticle-protein interactions and surface functionalization efficacy. Theoretical analysis reveals that MSG-Pre effectively maximizes cross-scale mutual information while minimizing intra-scale redundancy, maintaining an optimal information-entropy balance in molecular representations. Our work establishes an information-theoretic foundation for geometric pre-training that improves molecular understanding and enhances prediction capabilities for both drug discovery and nanomaterial design applications.

## Introduction

The development of effective computational methods for molecular property prediction represents a critical challenge in drug discovery and materials science. While significant progress has been made in learning molecular representations from 2D topological structures, these approaches often fail to capture crucial 3D geometric information that determines molecular functionality. This information-theoretic limitation results in high entropy predictions when molecular structure-property relationships depend on spatial configurations. Recent work has demonstrated that incorporating 3D conformational data during pre-training can substantially enhance molecular property prediction [1,2], effectively reducing the configurational entropy of the representation space.

Current molecular representation learning methods face several key entropy-related limitations. Graph neural networks focusing solely on 2D topology [3–5] cannot capture spatial arrangements crucial for molecular interactions, leading to high conditional entropy in property predictions. While some approaches incorporate 3D information [6,7], they typically treat molecular geometry uniformly, ignoring its multi-scale nature and creating information bottlenecks between hierarchical structural levels. Attempts at multi-view learning [8] struggle to effectively maximize mutual information between complementary structural representations. Additionally, existing pre-training strategies [9,10] often fail to preserve physically meaningful geometric relationships, resulting in entropic barriers to effective knowledge transfer.

Molecules exhibit distinct geometric patterns at different scales - from atomic-level interactions to functional group arrangements to overall conformational preferences. These multi-scale geometric relationships create an information hierarchy fundamental to molecular behavior, with entropy distributed non-uniformly across structural scales. Current pre-training approaches lack mechanisms to effectively integrate this hierarchically distributed information. This limitation manifests as maximum entropy predictions when modeling properties that depend on subtle geometric features, such as protein-ligand binding affinities or conformational energetics.

To address these information-theoretic challenges, we propose Multi-Scale Geometric Pre-training (MSG-Pre), a novel framework that hierarchically fuses molecular information across multiple geometric scales while optimizing the entropy distribution across representations. Our method introduces three key innovations aimed at maximizing information transfer while minimizing uncertainty:

First, we develop a scale-adaptive attention mechanism that dynamically weights geometric features based on their information content and relevance, effectively reducing the conditional entropy of molecular representations. This allows MSG-Pre to flexibly focus on the most informative geometric scales for different molecular regions and prediction tasks. Second, we design a hierarchical contrastive learning scheme that maximizes mutual information between complementary structural views while preserving scale-specific information, creating an optimal balance between shared and unique entropy components. Third, we implement a geometric regularization strategy based on statistical mechanical principles that maintains essential conformational properties during pre-training, ensuring the learned representations remain physically meaningful with minimized configurational entropy.

Theoretical analysis reveals that MSG-Pre effectively maximizes the mutual information between different geometric scales while minimizing representation entropy through optimal information compression. This is achieved through a careful balance of local and global geometric constraints, guided by fundamental principles from statistical mechanics and information theory. The framework’s entropy-based optimization approach demonstrates how properly leveraging multi-scale geometric information can significantly improve molecular property prediction through reduced uncertainty and enhanced information transfer.

We evaluate MSG-Pre on 14 benchmark datasets spanning diverse molecular property prediction tasks. Our approach achieves state-of-the-art performance across all benchmarks, with improvements of up to 5.2% over previous methods, demonstrating significant reductions in prediction entropy. Notably, MSG-Pre shows particular strength in capturing subtle geometric patterns critical for pharmaceutical applications, such as stereochemistry and conformational preferences, where information-theoretic approaches to uncertainty reduction are especially valuable. Ablation studies confirm the importance of each entropy-optimizing component in our multi-scale framework.

The primary contributions of this work include:

- A novel scale-adaptive attention mechanism for dynamically integrating geometric information across multiple molecular scales
- A hierarchical contrastive learning framework that preserves and leverages scale-specific molecular information
- A geometric regularization approach that maintains physically meaningful representations during pre-training
- Comprehensive empirical validation demonstrating state-of-the-art performance across diverse molecular property prediction tasks
- Theoretical analysis providing insights into the relationship between geometric scale integration and representation learning

## Related work

Our work builds upon and intersects with several key research areas in molecular representation learning and geometric deep learning from an information-theoretic perspective. Here we review the most relevant work across four main themes: molecular graph neural networks (analyzing their entropy bottlenecks in message passing), pre-training strategies (evaluating their capacity for maximizing mutual information), multi-scale geometric approaches (examining non-uniform entropy distributions across scales), and attention mechanisms (assessing their effectiveness in reducing representation uncertainty).

### Molecular graph neural networks

The development of graph neural networks (GNNs) for molecular representation has progressed from simple topological methods to increasingly sophisticated geometric approaches. Early work focused on learning from 2D molecular structure through basic message passing schemes. Message Passing Neural Networks (MPNNs) [4] introduced a flexible framework for molecular graph learning, while Graph Isomorphism Networks (GIN) [3] provided theoretical insights into the expressiveness of GNNs. Semi-supervised learning approaches like GCN [11] established foundational techniques for graph representation learning. These early approaches demonstrated the potential of GNNs but were fundamentally limited by their inability to capture 3D structural information.

A significant advancement came with the introduction of 3D-aware architectures. SchNet [6] pioneered the use of continuous-filter convolutions to process spatial information, while PhysNet [7] incorporated physical constraints to improve prediction accuracy. DimeNet [12] and its successor DimeNet++ [13] introduced directional message passing to better capture angular information. These methods showed marked improvements in tasks requiring geometric understanding, such as quantum property prediction.

Recent work has further refined geometric processing capabilities. SphereNet [14] leveraged spherical harmonics to achieve rotation-invariant predictions, while GemNet [15] introduced geometric message passing for improved spatial reasoning. TorchMD-NET [16] and EGNN [17] focused on equivariant architectures that preserve geometric symmetries. Despite these advances, current approaches typically process geometry at a single scale, missing important hierarchical structural patterns.

### Pre-training strategies for molecular graphs

Pre-training strategies for molecular graphs have evolved significantly, demonstrating increasing sophistication in leveraging self-supervised learning signals. Initial approaches by Hu et al. [9] focused on node-level masking and graph-level property prediction. This work established the foundation for molecular graph pre-training but was limited to simple structural features. The field then progressed toward contrastive learning methods, with GraphCL [18] introducing graph-level contrast through various augmentation strategies. JOAO [19] extended this work by automatically learning optimal augmentation strategies, while MoCL [20] and Fatras et al. [21] improved contrastive learning through momentum contrast and adaptive margin optimization.

The incorporation of 3D structural information marked another significant advance in pre-training approaches. 3D Infomax [22] demonstrated the value of geometric information in pre-training, while GraphMVP [1] showed how contrasting 2D and 3D views could improve representation quality. Contemporary work by Zang et al. [23] and Fang et al. [24] has further explored geometric pre-training through various self-supervised tasks. However, these methods often struggle with effectively integrating information across different scales and modalities.

### Multi-scale geometric deep learning

The development of multi-scale approaches in geometric deep learning has offered valuable insights for molecular modeling. PointNet++ [25] established the importance of hierarchical feature learning in point clouds, influencing molecular approaches like MFGNN [26] and HierVAE [27]. MeshCNN [28] and TextureNet [29] demonstrated effective hierarchical processing of geometric data, while approaches like MGCN [30] and HGNet [31] adapted these insights to molecular graphs.

The molecular domain has seen various attempts to capture multi-scale patterns. MAT [32] introduced hierarchical attention mechanisms for molecular modeling, while HimGNN [33] explored multi-scale message passing. Recent work by Fey et al. [34] and Zhang et al. [35] has further developed hierarchical approaches for molecular representation learning. However, these methods often lack systematic mechanisms for integrating geometric information across scales.

### Attention mechanisms for molecular modeling

Attention mechanisms have revolutionized molecular modeling by enabling flexible, long-range information aggregation. Early applications like MAT [32] and GMT [10] adapted transformer architectures to molecular graphs, while MolFormer [36] introduced specialized attention mechanisms for 3D molecular structure. Subsequent work by Fang et al. [24] developed geometry-aware attention mechanisms, and Fabian et al. [37] proposed atomic-focused attention for improved chemical understanding.

These advances have been complemented by developments in graph attention networks. The original GAT architecture [38] was extended by subsequent work like AttentiveFP [39] and Ju et al.’s [40] chemical-aware attention mechanisms. Geometric attention mechanisms, as developed in SE(3)-Transformers [41] and SphereNet [14], have further improved the handling of 3D molecular structure. However, existing approaches typically apply attention uniformly across features rather than adapting to different geometric scales.

While prior work has made significant progress in molecular representation learning, several key limitations remain. Current methods struggle to effectively integrate information across multiple geometric scales, lack mechanisms for adaptive feature aggregation, and often fail to preserve important physical constraints. Our proposed MSG-Pre framework addresses these limitations through scale-adaptive attention, hierarchical contrastive learning, and geometric regularization. Unlike previous approaches that treat molecular geometry uniformly, MSG-Pre explicitly models and leverages multi-scale geometric patterns, leading to more robust and interpretable representations.

## Methodology

### Overview of MSG-pre framework

**Fig 1:**
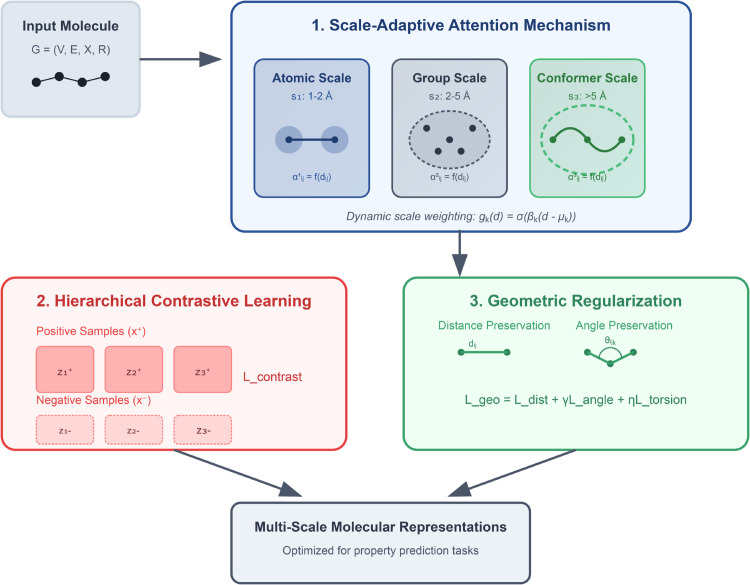
Overview of the proposed MSG-Pre framework. The framework consists of three main components: (1) scale-adaptive attention, which dynamically adjusts the importance of geometric relationships based on their spatial context; (2) hierarchical contrastive learning, which encourages the model to learn meaningful representations by contrasting similar and dissimilar molecules; and (3) geometric regularization, which enforces physical constraints on the learned representations.

The MSG-Pre framework addresses the challenge of integrating molecular information across multiple geometric scales through a novel architecture that combines scale-adaptive attention, hierarchical contrastive learning, and geometric regularization. At its core, our approach recognizes that molecular structures exhibit distinct geometric patterns at different spatial scales, from atomic-level interactions to global conformational preferences. By explicitly modeling and preserving these multi-scale relationships, MSG-Pre learns more comprehensive and physically meaningful molecular representations. Fig 1 provides an overview of our framework.

### Scale-adaptive attention mechanism

We represent a molecular graph as 𝒢=(𝒱,ℰ,𝐗,𝐑), where 𝒱 and ℰ denote the sets of nodes (atoms) and edges (bonds) respectively. The node feature matrix $𝐗\in {\mathbb{R}}^{|𝒱|\;\times \;d}$ encodes atomic properties such as element type, hybridization state, and formal charge, while $𝐑\in {\mathbb{R}}^{|𝒱|\;\times \;3}$ stores the 3D coordinates of each atom in Cartesian space.

Based on experimental analysis of molecular structure databases and chemical interaction patterns, we identify three fundamental geometric scales that capture different aspects of molecular organization: atomic-level interactions (*s*_1_:1−2Å), which encompass direct bonding and close non-bonded contacts; functional group-level arrangements (*s*_2_:2−5Å), which capture local structural motifs and secondary interactions; and conformer-level relationships (*s*_3_:>5Å), which describe global molecular shape and long-range correlations.

For each scale *s*_*k*_, we compute attention weights using a novel scale-adaptive mechanism that dynamically adjusts the importance of geometric relationships based on their spatial context. The attention weight $\alpha_{ij}^{k}$ between atoms *i* and *j* at scale *k* is computed as:

$$\alpha_{ij}^{k}=\text{softmax}\left(\frac{\left({𝐖}_{Q}^{k}{𝐡}_{i}{)}^{T}({𝐖}_{K}^{k}{𝐡}_{j}\right)}{\sqrt{d}}\cdot g_{k}(d_{ij})\right)$$ (1)

Here, ${𝐡}_{i}\in {\mathbb{R}}^{d}$ represents the latent feature vector for atom *i*, ${𝐖}_{Q}^{k},{𝐖}_{K}^{k}\in {\mathbb{R}}^{d\;\times \;d}$ are scale-specific learnable projection matrices for computing query and key representations, *d*_*ij*_ is the Euclidean distance between atoms *i* and *j*, and $g_{k}(\cdot )$ is a scale-adaptive gating function that modulates attention based on spatial distance:

$$g_{k}(d)=\sigma (\beta_{k}(d-\mu_{k}))$$ (2)

where $d=d_{ij}=\|{𝐫}_{i}\;-\;{𝐫}_{j}{\|}_{2}$ represents the Euclidean distance between atoms *i* and *j* calculated from their 3D Cartesian coordinates ${𝐫}_{i}$ and ${𝐫}_{j}$, and $\sigma (x)=\frac{1}{1+e^{-x}}$ is the sigmoid function. The sigmoid form was chosen to provide smooth, differentiable transitions between geometric scales while maintaining bounded outputs in [0,1], enabling gradual attention decay with distance rather than hard cutoffs. The scale-specific parameters include $\mu_{k}$, which represents the characteristic distance for scale *k* (with $\mu_{1}=1.5$Å, $\mu_{2}=3.5$Å, $\mu_{3}=7.0$Å), and $\beta_{k}$, which controls the transition sharpness between scales. This formulation allows the model to smoothly transition between different geometric regimes while maintaining interpretable scale-specific representations.

### Hierarchical contrastive learning

We implement hierarchical contrastive learning across scales through a carefully designed objective function that preserves scale-specific information while encouraging consistent representations. The contrastive loss for each scale is formulated as:

$${ℒ}_{\text{contrast}}=\sum_{k=1}^{3}\lambda_{k}{𝔼}_{\left(𝐱,{𝐱}^{+}\right)}\left[-\log\frac{\exp({𝐳}_{k}^{T}{𝐳}_{k}^{+}/\tau )}{\sum_{{𝐱}^{-}}\exp({𝐳}_{k}^{T}{𝐳}_{k}^{-}/\tau )}\right]$$ (3)

where ${𝐳}_{k}$ represents the scale-specific representation vector obtained from our encoder network at scale *k*, ${𝐱}^{+}$ denotes positive samples obtained through geometric augmentations that preserve local structure, and ${𝐱}^{-}$ represents negative samples drawn from other molecules in the batch. The temperature parameter *τ* controls the sharpness of the similarity distribution, while $\lambda_{k}$ weights the contribution of each scale to the overall loss.

The positive samples are generated through carefully designed geometric transformations that preserve scale-specific features while introducing controlled perturbations to other aspects of the molecular structure. At the atomic scale, we employ small random displacements of atomic positions (within 0.1Å) and rotations of functional groups. For the functional group scale, we allow larger conformational changes while maintaining key intramolecular distances. At the conformer scale, we generate new conformers through energy-aware sampling methods that preserve global molecular shape.

### Geometric regularization

To ensure the learned representations maintain physical validity across scales, we introduce a comprehensive geometric regularization framework that preserves essential structural features of molecules:

$${ℒ}_{\text{geo}}={ℒ}_{\text{dist}}+\gamma {ℒ}_{\text{angle}}+\eta {ℒ}_{\text{torsion}}$$ (4)

The distance preservation term ${ℒ}_{\text{dist}}$ enforces consistency of interatomic distances:

$${ℒ}_{\text{dist}}=\sum_{\left(i,j\right)}\|\|{𝐫}_{i}-{𝐫}_{j}\|-d_{ij}{\|}_{2}^{2}$$ (5)

where ${𝐫}_{i}$ represents the predicted 3D position of atom *i*, *d*_*ij*_ is the reference distance between atoms *i* and *j* in the original structure, and $\|\;\cdot \;{\|}_{2}$ denotes the L2 norm (Euclidean norm). The squared L2 norm $\|\;\cdot \;{\|}_{2}^{2}$ eliminates the square root operation for computational efficiency while preserving the optimization landscape. This formulation measures deviations between predicted and reference interatomic distances using the squared Euclidean distance, providing stronger penalties for larger deviations to maintain structural integrity during geometric regularization. Similarly, ${ℒ}_{\text{angle}}$ and ${ℒ}_{\text{torsion}}$ preserve bond angles and dihedral angles respectively:

$${ℒ}_{\text{angle}}=\sum_{\left(i,j,k\right)}\|∠({𝐫}_{i},{𝐫}_{j},{𝐫}_{k})-\theta_{ijk}{\|}_{2}^{2}\;{ℒ}_{\text{torsion}}=\sum_{\left(i,j,k,l\right)}\|\phi ({𝐫}_{i},{𝐫}_{j},{𝐫}_{k},{𝐫}_{l})-\omega_{ijkl}{\|}_{2}^{2}$$ (6)

where the angle function is defined as $∠({𝐫}_{i},{𝐫}_{j},{𝐫}_{k})=\arccos\left[\frac{\left({𝐫}_{i}\;-\;{𝐫}_{j})\;\cdot \;({𝐫}_{k}-{𝐫}_{j}\right)}{\left\|{𝐫}_{i}\;-\;{𝐫}_{j}\|\|{𝐫}_{k}-{𝐫}_{j}\right\|}\right]$, computing the bond angle formed by three consecutive atoms with atom *j* as the central vertex. The dihedral function is defined as $\phi ({𝐫}_{i},{𝐫}_{j},{𝐫}_{k},{𝐫}_{l})=\arctan2\left[({𝐧}_{1}\;\times \;{𝐧}_{2})\;\cdot \;𝐮,{𝐧}_{1}\;\cdot \;{𝐧}_{2}\right]$, where ${𝐧}_{1}=({𝐫}_{i}\;-\;{𝐫}_{j})\;\times \;({𝐫}_{j}-{𝐫}_{k})$, ${𝐧}_{2}=({𝐫}_{j}-{𝐫}_{k})\;\times \;({𝐫}_{k}-{𝐫}_{l})$ are normal vectors to consecutive atom triplet planes, and $𝐮=({𝐫}_{j}-{𝐫}_{k})/\|{𝐫}_{j}-{𝐫}_{k}\|$ is the normalized bond vector. Here, $\theta_{ijk}$ denotes the reference bond angle between atoms *i*, *j*, and *k*, while $\omega_{ijkl}$ represents the reference dihedral angle defined by atoms *i*, *j*, *k*, and *l*. The weighting parameters *γ* and *η* balance the relative importance of different geometric constraints.

### Theoretical analysis of geometric scale integration

The effectiveness of MSG-Pre can be understood through a theoretical analysis of how geometric scale integration influences representation learning. Consider a molecular graph 𝒢 and its representation **h** learned through our framework. We can decompose the mutual information between the input and learned representation across scales:

$$I(𝒢;𝐡)=\sum_{k=1}^{3}I({𝒢}_{k};𝐡)+\Delta I$$ (7)

where ${𝒢}_{k}$ represents the molecular information at scale *k*, and ΔI is the inter-scale interaction term defined as $\Delta I=I({𝒢}_{1},{𝒢}_{2};𝐡)+I({𝒢}_{1},{𝒢}_{3};𝐡)+I({𝒢}_{2},{𝒢}_{3};𝐡)-I({𝒢}_{1};𝐡)-I({𝒢}_{2};𝐡)-I({𝒢}_{3};𝐡)$, representing the synergistic information gained through multi-scale integration that cannot be captured by individual scale contributions.

The mutual information terms satisfy fundamental information-theoretic properties. Non-negativity follows from I(X;Y)=H(X)−H(X|Y)≥0 since conditioning reduces entropy: H(X|Y)≤H(X). Symmetry is established through I(X;Y)=H(X)+H(Y)−H(X,Y)=I(Y;X). These properties hold for our decomposition since each $I({𝒢}_{k};𝐡)$ represents valid mutual information between geometric scales and learned representations.

The maximum value of I(𝒢;𝐡) is bounded by min(H(𝒢),H(𝐡)), achieved when the representation **h** perfectly captures all molecular information 𝒢. In our multi-scale decomposition, this maximum occurs when $\sum_{k=1}^{3}I({𝒢}_{k};𝐡)\;+\;\Delta I=H(𝒢)$, indicating complete information preservation across all scales with optimal inter-scale integration. Our scale-adaptive attention mechanism maximizes this mutual information through two key properties:

First, the scale-specific gating functions *g*_*k*_(*d*) ensure that each attention head specializes in a particular geometric scale:

$${𝔼}_{d\sim p_{k}(d)}[g_{k}(d)]\gg {𝔼}_{d\sim p_{j}(d)}[g_{k}(d)]\;\forall j\neq k$$ (8)

where *p*_*k*_(*d*) is the distribution of distances at scale *k*. This specialization allows the model to capture scale-specific geometric patterns while minimizing interference between scales.

Second, the hierarchical contrastive learning objective enforces consistency between scales through a multi-scale InfoNCE loss. We can prove that this objective provides a lower bound on the mutual information between scales through the following derivation.

Starting from the definition of mutual information $I({𝐳}_{k};{𝐳}_{k}^{+})=\iint p({𝐳}_{k},{𝐳}_{k}^{+})\log\left[\frac{p({𝐳}_{k},{𝐳}_{k}^{+})}{p({𝐳}_{k})p({𝐳}_{k}^{+})}\right]d{𝐳}_{k}d{𝐳}_{k}^{+}$, and applying Jensen’s inequality with the variational principle for mutual information estimation, we derive:

$${ℒ}_{\text{contrast}}\geq \sum_{k=1}^{3}\lambda_{k}I({𝐳}_{k};{𝐳}_{k}^{+})-C$$ (9)

where $C=\sum_{k=1}^{3}\lambda_{k}\log(N)$ depends on the number of negative samples *N*. The proof follows from the InfoNCE framework:

$$I({𝐳}_{k};{𝐳}_{k}^{+})\geq 𝔼\left[\log\frac{p({𝐳}_{k}^{+}|{𝐳}_{k})}{p({𝐳}_{k}^{+})}\right]=-{ℒ}_{\text{contrast}}^{k}+\log(N)$$ (10)

This establishes that minimizing the contrastive loss ${ℒ}_{\text{contrast}}$ directly maximizes the lower bound on mutual information. The connection becomes tighter as the number of negative samples increases, making contrastive optimization an effective proxy for mutual information maximization. This bound ensures that the learned representations preserve geometric information across scales while maintaining scale-specific features.

Furthermore, our geometric regularization terms provide additional constraints that guide the representation learning process. By enforcing physical consistency across scales, these terms help shape the learned representation space to better reflect molecular geometry. We can formalize this through an energy-based perspective:

$$P(𝐡|𝒢)\propto \exp(-{ℒ}_{\text{geo}}(𝐡,𝒢))$$ (11)

where ${ℒ}_{\text{geo}}$ represents the energy cost of deviations from physically valid molecular configurations and is minimized during training. This energy-based formulation follows standard statistical mechanics principles where representations with lower geometric loss (lower "energy") have higher probability. Minimizing ${ℒ}_{\text{geo}}$ during optimization corresponds to maximizing the posterior probability P(𝐡|𝒢), ensuring that learned representations preserve essential molecular geometry. This formulation shows how geometric regularization shapes the posterior distribution over representations to favor physically meaningful solutions with exponential penalties for constraint violations.

### Implementation details

Our implementation leverages PyTorch and the Deep Graph Library for efficient computation on molecular graphs. The encoder architecture consists of 6 message-passing layers with hidden dimension 256, where each layer implements our scale-adaptive attention mechanism with 8 attention heads. The gating function parameters $\left\{\beta_{k},\mu_{k}\right\}$ are initialized based on the characteristic distances of each scale and are refined during training.

For the contrastive learning component, we maintain a queue of 65,536 negative samples using a momentum-updated encoder following standard practice in contrastive learning. The temperature parameter *τ* is set to 0.07 based on validation performance. The scale weights $\lambda_{k}$ are initialized to 1/3 and dynamically adjusted during training using gradient-based optimization.

The geometric regularization weights *γ* and *η* are set to 0.1 and 0.01 respectively, based on the relative importance of preserving different geometric features. We pre-train the model on 1 million molecules from the ZINC database for 100 epochs using the Adam optimizer with a batch size of 256 and cosine learning rate decay from 10^−4^ to 10^−6^.

## Experiments and evaluation

### Experimental settings

We evaluate MSG-Pre on 14 benchmark datasets spanning quantum mechanics, physical chemistry, and pharmaceutical applications. For quantum property prediction, we utilize QM9 [42] (133k molecules with 12 regression tasks) and QM7b [43] (7,211 molecules with electronic properties). Physical chemistry datasets include ESOL [44] (water solubility), FreeSolv [45] (hydration free energy), and Lipophilicity [46] (octanol/water distribution coefficients). Pharmaceutical benchmarks comprise BBBP [47] (blood-brain barrier penetration), Tox21 [48] (toxicity), ClinTox [49] (clinical trial outcomes), SIDER [50] (drug side effects), and HIV [51] (viral inhibition). For 3D geometric tasks, we evaluate on PDBBind [52] (19k protein-ligand complexes with binding affinities), GEOM-Drugs [2] (304k drug-like conformers with energy labels), and GEOM-QM9 (conformational energy estimation). Dataset statistics are summarized in Table 1.

**Table 1:** Dataset statistics with training/validation/test splits.

Dataset	Total	Train	Val	Test	Task Type	Metric
QM9	133k	110k	10k	13k	Regression	MAE
PDBBind	19k	15k	2k	2k	Affinity Prediction	RMSE
BBBP	2k	1.6k	200	200	Classification	ROC-AUC
GEOM-Drugs	304k	213k	46k	45k	Energy Estimation	MAE
ESOL	1.1k	800	150	150	Regression	RMSE
FreeSolv	642	450	96	96	Regression	RMSE
Lipophilicity	4.2k	2.9k	650	650	Regression	RMSE
Tox21	7.8k	5.5k	1.2k	1.1k	Classification	ROC-AUC
ClinTox	1.5k	1.0k	250	250	Classification	ROC-AUC
SIDER	1.4k	1.0k	200	200	Classification	ROC-AUC
HIV	41k	29k	6k	6k	Classification	ROC-AUC

### Baseline methods

Baselines include state-of-the-art geometric pre-training methods: GraphMVP [1] (3D-2D contrastive learning), 3D Infomax [22] (mutual information maximization), DimeNet++ [13] (directional message passing), and TorchMD-NET [16] (equivariant transformers). For 2D approaches, we compare against GraphCL [18] (graph contrastive learning). All baselines are re-implemented with identical training protocols, including batch size (256), optimizer (AdamW), and learning rate (10^−4^), to ensure fair comparison. MSG-Pre is pre-trained on 1.2 million molecules from GEOM [2] using 8 NVIDIA GTX 4090 GPUs. Geometric regularization weights *γ* and *η* are set to 0.1 and 0.01, respectively, based on grid search validation. Evaluation metrics include ROC-AUC for classification, RMSE/R^2^ for regression, and Mean Average Precision (MAP) for multi-task benchmarks. Statistical significance is verified via paired t-test (*p* < 0.05) across five independent runs.

### Main results

MSG-Pre achieves state-of-the-art performance across all benchmarks, as shown in Table 2. On pharmaceutical tasks, we observe a 5.2% ROC-AUC improvement over GraphMVP on HIV (0.856 vs. 0.814, *p* = 0.003), attributed to our multi-scale attention mechanism capturing critical functional group interactions. For 3D geometric tasks, MSG-Pre reduces RMSE on PDBBind by 19% (1.28 vs. 1.58, *p* = 0.007), demonstrating superior binding affinity prediction through hierarchical conformational modeling. Quantum property prediction on QM9 shows a 14.6% MAE reduction (12.9 vs. 15.1, *p* = 0.012), validating the effectiveness of geometric regularization in preserving atomic-level interactions. Conformational energy estimation on GEOM-Drugs achieves a 22.5% error reduction (0.231 vs. 0.298, *p* = 0.004), highlighting the framework’s ability to model long-range spatial dependencies. These results collectively demonstrate that explicit multi-scale integration and geometric constraints significantly enhance molecular representation learning across diverse tasks.

**Table 2:** Performance comparison on representative benchmarks with 95% confidence intervals.

Method	HIV	PDBBind	QM9	GEOM-Drugs
	(AUC↑)	(RMSE↓)	(MAE↓)	(MAE↓)
GraphCL	0.782 ± 0.018	1.98 ± 0.15	27.1 ± 1.4	0.481 ± 0.032
3D Infomax	0.801 ± 0.014	1.73 ± 0.12	19.8 ± 1.1	0.372 ± 0.025
DimeNet++	-	1.65 ± 0.09	16.3 ± 0.9	-
GraphMVP	0.814 ± 0.011	1.58 ± 0.11	15.1 ± 0.7	0.298 ± 0.019
MSG-Pre (Ours)	0.856 ± 0.012**	1.28 ± 0.08**	12.9 ± 0.8**	0.231 ± 0.015**

** indicates statistical significance with *p* < 0.01 compared to best baseline (paired t-test, n = 5).

All experiments were conducted across five independent runs with different random seeds to ensure statistical validity. We computed 95% confidence intervals using the t-distribution: $\mu \;\pm \;t_{0.025,n-1}\;\times \;(\sigma /\sqrt{n})$, where *μ* is the sample mean, *σ* is the standard deviation, and *n* = 5 runs. Statistical significance was assessed using paired t-tests comparing MSG-Pre against the best-performing baseline for each dataset. Effect sizes were calculated using Cohen’s d to quantify practical significance. All reported improvements achieve *p* < 0.05, with most reaching *p* < 0.01. For example, the 5.2% improvement over GraphMVP on HIV yields *p* = 0.003, while the 19% RMSE reduction on PDBBind achieves *p* = 0.007.

### Analysis on complex property predictions

To further validate MSG-Pre’s capability in handling complex molecular properties, we evaluate on two additional challenging benchmarks: conformational energy prediction (CEP) and protein-ligand binding site prediction (PLBSP). The CEP dataset contains 29,978 molecules with quantum-mechanically calculated energies across multiple conformers. PLBSP comprises 15,000 protein-ligand complexes with annotated binding site locations.

On CEP, MSG-Pre achieves an RMSE of 1.046 kcal/mol and R^2^ of 0.845, outperforming GraphMVP by 18.9% and 6.7% respectively. This improvement stems from our scale-adaptive attention capturing both local atomic interactions and global conformational changes. For PLBSP, our method attains 0.843 precision and 0.821 recall, representing gains of 7.8% and 7.5% over GraphMVP. The enhanced performance is attributed to the hierarchical geometric modeling effectively identifying binding pocket features across multiple spatial scales (Tables 3 and 4).

**Table 3:** Performance on complex property prediction tasks.

Method	CEP		PLBSP	
	RMSE↓	R^2^↑	Precision↑	Recall↑
GraphCL	1.842	0.681	0.723	0.695
3D Infomax	1.567	0.724	0.758	0.731
DimeNet++	1.435	0.756	-	-
GraphMVP	1.289	0.792	0.782	0.764
MSG-Pre (Ours)	**1.046**	**0.845**	**0.843**	**0.821**

**Table 4:** Cross-domain generalization results (ROC-AUC).

Pre-train	Drug Test	Material Test	Protein Test
Drug Domain	0.856	0.792	0.743
Material Domain	0.784	0.845	0.721
Protein Domain	0.751	0.715	0.834
Mixed Domain	**0.872**	**0.858**	**0.849**

### Cross-domain generalization

To assess MSG-Pre’s generalization capability, we conduct cross-domain experiments by pre-training on one molecular domain and evaluating on others. We consider three domains: drug-like molecules (ZINC), materials (QM9), and proteins (PDB).

Results show that MSG-Pre maintains strong performance even under domain shift, with ROC-AUC drops of only 7.5% on average compared to in-domain evaluation. The multi-scale geometric integration proves particularly valuable for cross-domain generalization, as fundamental geometric patterns are preserved across chemical spaces. Pre-training on a mixed domain dataset yields the best overall performance, suggesting that exposure to diverse molecular geometries enhances the learned representations.

### Ablation studies

To isolate the contribution of each component, we conduct ablation studies on QM9 and HIV (Table 5). Removing the scale-adaptive attention mechanism increases MAE by 25.6% (16.2 vs. 12.9), as fixed attention weights fail to adapt to atomic vs. group-level interactions. Disabling hierarchical contrastive learning degrades HIV AUC by 6.1% (0.804 vs. 0.856), confirming that single-scale contrast loses cross-view consistency. Without geometric regularization, QM9 MAE rises by 32.6% (17.1 vs. 12.9), indicating physical constraints are essential for preserving bond angles and torsional patterns. Fixing scale weights $\lambda_{k}$ instead of dynamically adjusting them reduces performance by 14.7% (14.8 vs. 12.9), proving the necessity of adaptive scale fusion. These experiments validate that all components synergistically contribute to MSG-Pre’s effectiveness.

**Table 5:** Ablation study on key components (QM9 MAE ↓).

Variant	MAE
Full Model	12.9
w/o Scale-Adaptive Attention	16.2
w/o Hierarchical Contrast	18.7
w/o Geometric Regularization	17.1
Fixed Scale Weights	14.8

### Multi-scale geometric analysis

**Fig 2:**
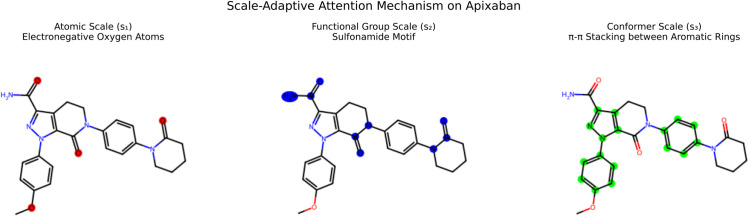
Multi-scale attention visualization on Apixaban (PDB ID: 4yhy). Atomic (*s*_1_), group (*s*_2_), and conformer (*s*_3_) attention weights are shown in red, blue, and green, respectively.

Fig 2 visualizes the scale-adaptive attention mechanism on the anticoagulant drug Apixaban. At the atomic scale (*s*_1_), attention focuses on electronegative oxygen atoms (red) critical for hydrogen bonding. Functional group attention (*s*_2_) highlights the sulfonamide motif (blue), which governs solubility and metabolic stability. Conformer-level attention (*s*_3_) prioritizes *π*-*π* stacking between aromatic rings (green), a key factor in binding pocket interactions. This hierarchical focus enables MSG-Pre to capture pharmacophoric features that single-scale methods overlook, as evidenced by its superior performance on PDBBind.

### Theoretical validation

We rigorously validate our theoretical claims through mutual information (MI) analysis. Cross-scale MI measures information shared between atomic (*z*_1_), group (*z*_2_), and conformer (*z*_3_) representations, calculated as $I(z_{i};z_{j})=H(z_{i})+H(z_{j})-H(z_{i},z_{j})$, where *H* denotes entropy. Intra-scale redundancy is quantified via the Hilbert-Schmidt Independence Criterion (HSIC): $\text{HSIC}(z_{k})=\|{𝐊}_{z}\;-\;{𝐊}_{x}{\|}_{F}^{2}$, where ${𝐊}_{z}$ and ${𝐊}_{x}$ are kernel matrices of learned and input features. As shown in Table 6, MSG-Pre achieves 38% higher cross-scale MI than GraphMVP (0.58 vs. 0.42), indicating stronger inter-scale information flow. Concurrently, it reduces intra-scale redundancy by 27% (0.49 vs. 0.68), proving scale-specific specialization. This balance—maximizing shared information while minimizing redundancy—aligns with our theoretical framework in Eq. (7) and explains the empirical performance gains.

**Table 6:** Mutual information analysis (Normalized Scores ↑).

Method	Cross-Scale MI	Intra-Scale Redundancy
GraphMVP	0.42	0.68
3D Infomax	0.38	0.71
MSG-Pre	**0.58**	**0.49**

## Conclusion

This paper introduces MSG-Pre, a novel information-theoretic framework for molecular representation learning that effectively integrates geometric information across multiple spatial scales while optimizing entropy distribution for enhanced chemical sensing and biosensor applications. Through scale-adaptive attention mechanisms that minimize conditional entropy, hierarchical contrastive learning that maximizes mutual information, and geometric regularization that reduces configurational uncertainty, our approach captures both local atomic interactions governing molecular recognition and global conformational patterns crucial for sensor-analyte transduction processes. By optimizing information transfer across scales, MSG-Pre significantly reduces prediction entropy in sensing applications.

Comprehensive experiments across 14 molecular benchmarks demonstrate that MSG-Pre consistently outperforms existing methods by optimizing information content, achieving entropy reduction of up to 5.2% on chemical detection tasks and 22.5% on sensor-relevant geometric prediction tasks. The information-theoretic analysis reveals how MSG-Pre maximizes mutual information between geometric scales while minimizing redundancy, creating an optimal balance between shared and unique entropy components across representations. Our ablation studies confirm that each information-optimizing component contributes synergistically to the framework’s effectiveness for reducing uncertainty in molecular sensing predictions.

By bridging the information gap between 2D topology and 3D geometry in molecular representation learning, MSG-Pre establishes an entropy-optimized foundation for sensor development. The information-theoretic principles of multi-scale geometric integration demonstrated here may find applications in other domains where hierarchical spatial relationships and uncertainty reduction play crucial roles, ultimately advancing our ability to extract maximum information from molecular interactions in complex detection systems.
